# Knowledge, Attitude and Practice Regarding Risk of Diabetic Foot Among Diabetic Patients in Aseer Region, Saudi Arabia

**DOI:** 10.7759/cureus.18791

**Published:** 2021-10-14

**Authors:** Ali M Al Amri, Ibarhim M Shahrani, Yazan A Almaker, Daher M Alshehri, Mohammed A Argabi, Fouad A Alghamidi, Yahya Z Alqahtani

**Affiliations:** 1 Family Medicine & Diabetes, Asir Central Hospital, Abha, SAU; 2 College of Medicine, King Khalid University, Abha, SAU; 3 Surgery, Asir Central Hospital, Abha, SAU

**Keywords:** foot ulcers, awareness, attitude, practice, knowledge, care, diabetic foot, complications, diabetes mellitus

## Abstract

Background

Diabetic foot care is vital as it is preventable complication but dangerous even a small trauma can end with serious consequences. Diabetes may cause nerve damage that affects feet sensation. Diabetes may also reduce blood flow to the feet, making it harder to heal an injury or resist infection. Because of these problems, patients may lose notice of early foot abnormalities. Diabetic patients’ awareness regarding how to care for their feet plays a significant role in preventing these complications.

Methods

A correlation cross-sectional study was conducted targeting all diabetic patients aging 20 years or more. Online questionnaire was used for data collection. Questionnaire was uploaded online using social media platforms by the researchers and their relatives and friends. The questionnaire included patient personal data, patients’ knowledge regarding diabetic foot, attitude and practice regarding the risk of diabetic foot among diabetic patients.

Results

A total of 1,000 diabetic patients fulfilling the inclusion criteria completed the study questionnaire. Exact of 77.5% of the patients know that Diabetics can get gangrene in the foot, 74.9% know diabetics can develop ulcers in the foot, 66.7% reported that Diabetes can reduce blood flow to the feet of diabetic patients, 66.6% agreed that diabetic patients can suffer from a lack of sensation in the feet. Exact of 84.5% of the study participants agreed that Diabetics should check for any wounds on their feet daily, 78.7% agreed that Diabetics should visit a doctor when there is any infection or wound in the feet, 76.6% think that diabetic patients should wear specialized shoes to avoid diabetic foot according to the doctor's instructions. A total of 822 (82.2%) of the study patients wash their feet daily, and 295 (29.5%) usually wear cotton socks regularly and 39.6% sometimes wear the stock. Only 192 (19.2%) regularly walk barefoot and 41.7% sometimes do.

Conclusion

In conclusion, the current study results showed that nearly two out of each three diabetic patients were knowledgeable for diabetic foot and its care. High knowledge was associated with young age, high education and having family member with diabetes mellitus (DM). Also, patients had a good attitude towards diabetic foot care and the effect of diabetes on foot health with to some level accepted practice except for some issues.

## Introduction

Diabetes mellitus (DM) is a worldwide public health problem. with chronic progressive metabolic disorder featured by hyperglycaemia, caused by either deficiency or resistance to the insulin hormone [1,2]. A diabetic foot is one of the diabetes complications caused due from peripheral arterial disease (PAD) with sensory neuropathy at diabetic patients’ feet [3,4]. Diabetic foot is a long-lasting complication of diabetes with a high burden that affects diabetic patients’ quality of life [5-7]. Presence of several characteristic diabetic foot pathologies such as infection, diabetic foot ulcer and neuropathic osteoarthropathy is called diabetic foot syndrome [8].

There are many risk factors of the diabetic foot include old age, duration of diabetes, and hypertension. Peripheral neuropathy associated with sensory loss and peripheral vascular disease-resulting ischemia are the leading risk factors causing foot ulcer and eventually foot amputation [9-11]. And diabetic foot ulcers are frequent and most disturbing preventable complications of patients with long-term uncontrolled diabetes mellitus [12]. Diabetic foot ulcer-associated morbidity and early mortality because of chronic complications of foot was recorded. [11,12]. The most reported sites of these ulcers are the areas of the foot that encounters frequent trauma and pressure sensations [13]. Most of the diabetic foot complications can be prohibited with alert foot care. It takes effort and time to build up good foot care habits, but self-care is essential [14,15].

Diabetic patients’ awareness regarding how to care for their feet plays a significant role in preventing these complications with decreased social, medical, and economic burden of its consequences including foot ulcers and amputation. The current study aimed to assess the knowledge, attitudes, practices and risk factors influencing diabetic foot ulcers among diabetes patients in Aseer region, Southern of Saudi Arabia.

## Materials and methods

Methodology

A correlation cross-sectional study was conducted targeting all diabetic patients aging 20 years or more. Patients less than 20 years, Patient who is already having diabetic foot, amputated foot, or foot ulcers, and those who did not fill the study questionnaire were excluded. After having ethical approval, and due to the current environment due to COVID-19 pandemic, online questionnaire was used for data collection. All accessible adults in the general population were invited to fill the uploaded questionnaire consecutively till achieving the required sample size. The questionnaire was developed by researchers after intensive literature reviews and expert's consultation. Study questionnaire validity was assessed by a panel of three experts in diabetology with applying all confirmed modifications. Also, reliability and clarity were assessed using pilot of 30 diabetic patients who were excluded from the main study with α-Cronbach’s of 0.74. The questionnaire included patient personal data, patients’ knowledge regarding diabetic foot, attitude and practice regarding the risk of diabetic foot among diabetic patients. Questionnaire was uploaded online using social media platforms by the researchers and their relatives and friends.

Data analysis

After data were extracted, it was revised, coded, and fed to statistical software IBM SPSS version 22(SPSS, Inc. Chicago, IL). All statistical analysis was done using two-tailed tests. p-value less than 0.05 was statistically significant. For knowledge items, each correct answer was scored one point and the total summation of the discrete scores of the different items was calculated. A patient with a score less than 60% of the total score was considered to have poor awareness while good awareness was considered if he had a score of 60% of the total or more. Descriptive analysis based on frequency and percent distribution was done for all variables including participants' age, gender, education level, family history of diabetes. Also, participants’ knowledge items regarding diabetic foot, attitude towards diabetic foot care, and patients practice towards their foot were displayed. Crosstabulation was used to assess the distribution of knowledge level according to participants’ personal data and the distribution of patients practice according to their knowledge level. Relations were tested using Pearson chi-square test.

The study was approved by Regional Committee for Research Ethics, Directorate Health Affairs-Aseer Region with a study number of (REC-02-09-202). After the institutional review board (IRB) approval, the data was collected only by authorized members.

## Results

A total of 1,000 diabetic patients fulfilling the inclusion criteria completed the study questionnaire. Patients ages ranged from 20 to 59 years old with a mean age of 42.6 ± 11.8 years old. Exact of 670 (67%) of the patients were males and 658 (65.8%) were married. As for educational level, 566 (56.6%) were university graduated and 238 (23.8%) had secondary level of education. Exact of 678 (67.8%) patients had family history of diabetes (Table 1).

**Table 1:** . Personal data of diabetic patients, Aseer region, Saudi Arabia.

Personal data	No	%
Age in years		
20-40	391	39.1%
41-50	348	34.8%
> 50	261	26.1%
Gender		
Male	670	67.0%
Female	330	33.0%
Marital status		
Single	342	34.2%
Married	658	65.8%
Educational level		
Below secondary	196	19.6%
Secondary	238	23.8%
University/above	566	56.6%
Does anyone in your family have diabetes?		
Yes	678	67.8%
No	322	32.2%

Exact of 77.5% of the patients know that diabetics can get gangrene in the foot, 74.9% know diabetics can develop ulcers in the foot, 66.7% reported that diabetes can reduce blood flow to the feet of diabetic patients, 66.6% agreed that diabetic patients can suffer from a lack of sensation in the feet, and 51.4% know smoking can reduce blood flow to the feet (Table 2).

**Table 2:** Distribution of knowledge regarding diabetic foot care among diabetic patients, Aseer region, Saudi Arabia.

Knowledge item	Yes		No		Dont know	
	No	%	No	%	No	%
Diabetes can reduce blood flow to the feet of diabetic patients?	667	66.7%	80	8.0%	253	25.3%
Diabetic patient can suffer from a lack of sensation in the feet?	666	66.6%	113	11.3%	221	22.1%
Diabetics can develop ulcers in the foot?	749	74.9%	98	9.8%	153	15.3%
Diabetics can get gangrene in the foot?	775	77.5%	95	9.5%	130	13.0%
Smoking can reduce blood flow to the feet?	514	51.4%	132	13.2%	354	35.4%

**Figure 1:**
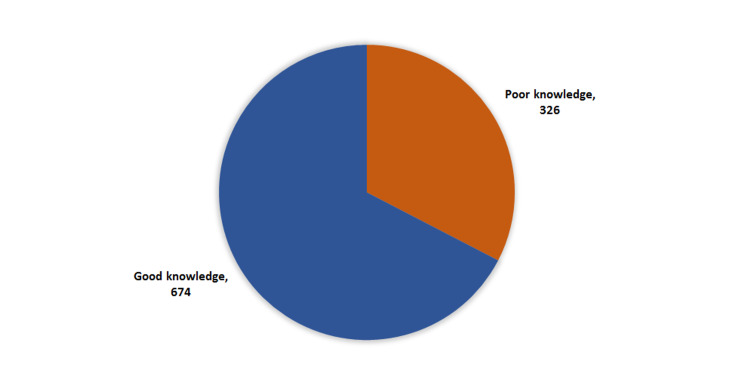
Overall knowledge regarding diabetic foot among diabetic patients, Aseer region, Saudi Arabia.

Exact of 674 (67.4%) patients had a good knowledge level regarding diabetic foot, while 326 (32.6%) had poor knowledge level (Figure 1).

Exact of 84.5% of the study participants agreed that diabetics should check for any wounds on their feet daily, 78.7% agreed that diabetics should visit a doctor when there is any infection or wound in the feet, 76.6% think that diabetic patients should wear specialized shoes to avoid diabetic foot according to the doctor's instructions. Only 20.9% agreed that diabetics should not make periodic visits to the diabetes clinic for the purpose of examination and 16.5% believe that I can treat myself if I have diabetes without consulting a doctor (Table 3).

**Table 3:** Distribution of attitude towards diabetic foot care among diabetic patients, Aseer region, Saudi Arabia.

Attitude items	Disagree		Agree		Unsure	
	No	%	No	%	No	%
Diabetics should check for any wounds on their feet daily	72	7.2%	845	84.5%	83	8.3%
Diabetics should visit a doctor when there is any infection or wound in the feet	93	9.3%	787	78.7%	120	12.0%
Diabetic patients should wear specialized shoes to avoid diabetic foot according to the doctor's instructions	110	11.0%	766	76.6%	124	12.4%
Diabetics should not make periodic visits to the diabetes clinic for the purpose of examination	693	69.3%	209	20.9%	98	9.8%
I believe that I can treat myself if I have diabetes without consulting a doctor	660	66.0%	165	16.5%	175	17.5%

A total of 822 (82.2%) of the study patients wash their feet daily, and 295 (29.5%) usually wear cotton socks regularly and 39.6% sometimes wear the stock. Only 192 (19.2%) regularly walk barefoot and 41.7% sometimes do. Changing shoes when damaged only is reported among 47% of the patients while 35.9% change the shoes more than once a year. Checking foot for any deformity was done monthly among 8.3% of the patients while 11.8% do it every six months, 11.3% do every year and 68.6% seek care when sick only. Exact of 70.4% of the patients reported that they Consult a physician when found any deformity (wound/ulcer) in their feet (Table 4).

**Table 4:** Distribution of diabetic patients practice regarding diabetic foot care, Aseer region, Saudi Arabia.

Practice items	No	%
Do you wash your feet daily?		
Yes	822	82.2%
Sometimes	111	11.1%
No	67	6.7%
Do you wear cotton socks regularly?		
Yes	295	29.5%
Sometimes	396	39.6%
No	309	30.9%
Do you always walk barefoot?		
Yes	192	19.2%
Sometimes	417	41.7%
No	391	39.1%
How often do you change your shoes?		
Once per year	171	17.1%
More than once pr year	359	35.9%
When the shoe is damaged	470	47.0%
How often do you go to check your feet?		
Monthly	83	8.3%
Every 6 months	118	11.8%
Every year	113	11.3%
When I am sick	686	68.6%
What do you do if you find deformities (wounds/ulcers) on your feet?		
Consult a physician	704	70.4%
Treat by yourself	296	29.6%

Good knowledge was detected among 71.6% of young age patients compared to 59.4% of old age group with recorded statistical significance (p=0.004). Also, 76.3% of the diabetic patients were university graduated versus 44.4% of those with below secondary education (p=0.001). Exact of 69.2% of patients with a family history of diabetes had good knowledge regarding diabetic foot care in comparison to 63.7% of those without (p=0.049) (Table 5).

**Table 5:** Distribution of diabetic patients’ overall knowledge regarding diabetic foot by their personal data. P: Pearson X^2^ test. *p < 0.05 (significant).

Personal data	Knowledge level				p-value
	Poor		Good		
	No	%	No	%	
Age in years					0.004*
20-40	111	28.4%	280	71.6%	
41-50	109	31.3%	239	68.7%	
> 50	106	40.6%	155	59.4%	
Gender					0.177
Male	209	31.2%	461	68.8%	
Female	117	35.5%	213	64.5%	
Marital status					0.102
Single	123	36.0%	219	64.0%	
Married	203	30.9%	455	69.1%	
Educational level					0.001*
Below secondary	109	55.6%	87	44.4%	
Secondary	83	34.9%	155	65.1%	
University/above	134	23.7%	432	76.3%	
Does anyone in your family have diabetes?					0.049*
Yes	209	30.8%	469	69.2%	
No	117	36.3%	205	63.7%	

Exact of 88.6% of diabetic patients with good knowledge level was their feet daily compared to 69% of others with poor knowledge (p=0.001). Also, 35.3% of diabetic patients with good knowledge wear cotton socks regularly versus 17.5% of those with poor knowledge (0.001). Exact of 14.5% of patients with good knowledge change their shoes annually versus 22.4% of those with poor knowledge (p=0.008) (Table 6).

**Table 6:** Diabetic patients’ overall knowledge regarding diabetic foot and their practice level. P: Pearson X^2^ test. *p < 0.05 (significant).

Practice	Knowledge level				p-value
	Poor		Good		
	No	%	No	%	
Do you wash your feet daily?					0.001*
Yes	225	69.0%	597	88.6%	
Sometimes	61	18.7%	50	7.4%	
No	40	12.3%	27	4.0%	
Do you wear cotton socks regularly?					0.001*
Yes	57	17.5%	238	35.3%	
Sometimes	158	48.5%	238	35.3%	
No	111	34.0%	198	29.4%	
Do you always walk barefoot?					0.379
Yes	62	19.0%	130	19.3%	
Sometimes	127	39.0%	290	43.0%	
No	137	42.0%	254	37.7%	
How often do you change your shoes?					0.008*
Once per year	73	22.4%	98	14.5%	
More than once pr year	107	32.8%	252	37.4%	
When the shoe is damaged	146	44.8%	324	48.1%	
How often do you go to check your feet?					0.001*
Monthly	32	9.8%	51	7.6%	
Every 6 months	46	14.1%	72	10.7%	
Every year	53	16.3%	60	8.9%	
When I am sick	195	59.8%	491	72.8%	
What do you do if you find deformities (wounds/ulcers) on your feet?					0.506
Consult a physician	225	69.0%	479	71.1%	
Treat by yourself	101	31.0%	195	28.9%	

## Discussion

The current study aimed to assess knowledge level of diabetic patients in Aseer region regarding diabetic foot and its care. Also, to identify their attitude towards diabetic foot care and their daily life practice regarding their foot care. Diabetic foot is one of the main complications of DM which diagnosed among nearly half of diabetic patients and causing about 80% of lower limb amputation [16,17]. Diabetic foot includes some type of neuropathy and angiopathy affecting mainly the foot ending with tissue damage, infection, and ulceration [9]. Poor diabetic control, long duration of uncontrolled diabetes, peripheral vasculopathy, older age, and poor awareness regarding diabetes generally and foot care particularly are the most known risk factors for diabetic foot [18].

In Saudi Arabia, diabetic foot ulcer is the leading cause of lower limb amputation, reference to the last few years, it caused lower limb amputation for 49.6% of cases [19] and it is predictable that this rate will show more increase next few years [20,21]. Diabetic foot in Saudi Arabia prevalent in 3.3% of diabetic patients; with 2.1% suffered foot ulcer and 1.1% had amputation [11]. Diabetic foot complications are preventable relative to all other diabetic complications [22,23]. Awareness of patients about foot care is vital to avoid diabetic foot consequences, and amputation [24]. Foot complications including foot ulcers are more frequent among diabetic patients with poor awareness of their foot care [25].

The current study showed that more than two-thirds of the diabetic patients were knowledgeable regarding diabetic foot and its care. The highest awareness was for diabetic foot consequences (ulcers and gangrene) and they were aware of the effect of diabetes on foot blood supply and neuropathy associated with lack of sensation. Knowledge level was significantly higher among young aged patients which may be due to their worry of diabetes-related complications which may affect their future life, highly educated patients who can read, explore and had related information by their own selves, and those with a family history of DM. Literature review showed high variety in estimated diabetic patients’ knowledge and awareness regarding diabetic foot and its care. This high variety of estimated awareness levels may be attributed to many factors including the method of awareness assessment, difference in samples characteristics and even the medical care strategies applied for diabetic patients in each country regarding doctor visits or ability to catch service and information through telephone. Solan YM et al. [26] found that 53.6% of diabetic patients reported a good knowledge level regarding diabetic foot. Males were more complaint to foot drying by 65.2%, while females are applying more attention to softening of skin by 72.3%. In Tanzania, Chiwanga FS et al. [27] showed that the mean knowledge score for diabetic foot was 11.2 out of a total possible score of 23 (48.6%). Low scores were reported among patients with low education (8.3 ± 6.1), diabetes duration of < 5 years (10.2 ± 6.7) and not receiving advice on foot care (8.0 ± 6.1). Among the 404 patients, 48 % had received advice on foot care, and 27.5 % had their feet examined by a doctor at least once since their initial diagnosis. Foot self-care was significantly higher in patients who had received advice on foot care and in those whose feet had been examined by a doctor at least once. Locally, in Saudi Arabia, Algshanen MA [28] reported that 55.1% of diabetic patients had high score for diabetic foot knowledge. About 69% had very low score 0-2 out of 6 in the assessment of previous education of diabetic foot. More than half of the participants (56.5%) had a score 6-10 out of 15 in the evaluation of practice with diabetic foot. Alhuqayl AA et al. [29] found that 53.3%, of diabetic patients had good knowledge about diabetic foot while low knowledge represented 46.7% among participants. The significant determinants for god knowledge level included patients gender (p < 0.001), education level (p = 0.001), DM type (p = 0.002), and suffering diabetic retinopathy (p = 0.009).

As for patients’ attitude and practice, the current study showed that the vast majority of the study patients agreed that diabetics should check for any wounds on their feet daily, and that they should visit a doctor when there is any infection or wound in the feet. More than three-quarters of the patients think that diabetic patients should wear specialized shoes to avoid diabetic foot according to the doctor's instructions. Only 20.9% agreed that Diabetics should not make periodic visits to the diabetes clinic for the purpose of examination and 16.5% believe that I can treat myself if I have diabetes without consulting a doctor. As for practice, more than 80% of the study patients wash their feet daily, but about half of them wear cotton socks. About half of the patients may walk barefoot which is unsafe due to the higher risk of trauma. Checking foot when had any problem was the most reported behaviour (68.6%). Exact of 70.4% of the patients reported that they consult a physician when found any deformity (wound/ulcer) in their feet.

## Conclusions

In conclusion, the current study results showed that nearly two out of each three diabetic patients were knowledgeable for diabetic foot and its care. High knowledge was associated with young age, high education and having family member with DM. Also, patients had a good attitude towards diabetic foot care and the effect of diabetes on foot health. Practice regarding patients’ foot was satisfactory except for avoiding trauma leading causes including walking with bared feet and change shoes with damage only not periodically. Also, feet check frequency was not high among the patients. Diabetic patients need periodic health education programs and training for all disease-related complications including foot-related consequences.
